# Antioxidant Capacity of Thyme (*Thymus vulgaris*) Essential Oil and Its Effect on In Vivo Fertility of Rams Subjected to Testicle Heat Stress

**DOI:** 10.1111/jpn.14063

**Published:** 2024-10-28

**Authors:** Samia Khnissi, Imène Ben Salem, Bochra Bejaoui, Sami Fattouch, Souha ben Mustapha, Rami Haj‐Kacem, Naceur M'Hamdi, Patrick Martin, Maria Dattena, Narjess Lassoued

**Affiliations:** ^1^ Laboratory of Animal and Forage Production National Institute of Agronomic Research of Tunisia (INRAT) University of Carthage Tunis Tunisia; ^2^ Department of Animal Production, Service of Zootechnics and Agricultural Economy National School of Veterinary Medicine Sidi Thabet University of Manouba Cité Nasr Tunisia; ^3^ Laboratory of Useful Materials National Institute of Research and Pysico‐Chemical Analysis (INRAP), Technopark of Sidi Thabet Ariana Tunisia; ^4^ Department of Chemistry University of Carthage, Faculty of Sciences of Bizerte Bizerte Zarzouna Tunisia; ^5^ EcoChemistry Laboratory, National Institute of Applied Sciences and Technology (INSAT) University of Carthage Carthage Tunisia; ^6^ Tunisia Polytechnic School, LEGI University of Carthage Carthage Tunisia; ^7^ Research Laboratory of Ecosystems and Aquatic Resources, National Agronomic Institute of Tunisia Carthage University Tunis Tunisia; ^8^ Unité Transformations and Agroressources, ULR7519 Université d'Artois‐UniLaSalle Béthune France; ^9^ Department of Animal Science Agricultural Research Agency of Sardinia Olmedo Sassari Italy

**Keywords:** antioxidant, heat stress, ram fertility

## Abstract

The detrimental effects of hyperthermia on the testes and the protective effect of thyme essential oil against testicular damage induced by this stress in rams were studied. Twenty‐four rams of the Barbarine breed with an average weight of 62.5 ± 0.3 kg and an average age of 24 ± 0.6 months. The experiment consisted of inducing localized heat stress on the first group of rams by applying heat bags to both testicles of six rams (G s). The second group underwent the same heat stress on the testes but received orally 100 µL/day/animal of thyme essential oil (G s‐he). A positive control did not undergo stress but received thyme essential oil (G he) with the same doses as the (G s‐he) group, and the negative control did not undergo either stress or receive the essential oil of thyme (G c). One hundred twenty‐eight adult ewes of the same breed divided into four groups of 32 ewes were used to study the effect of different treatments on the in vivo ram's fertility. Ewes are synchronized and we have applied natural mating with oestrus control, the reproduction balance sheet is calculated after lambing. The results showed that tests of heat stress (HS) negatively affect semen quality but did not cause infertility. However, neither tests for heat stress nor treatment with thyme EO significantly affected the haematological profile. The study of the effect of heat stress on the testes on fertility in vivo showed a drop in the number of females who were fertilized at the first oestrus and consequently a drop in fertility. However, the rams that suffered the same stress but were treated with EO thymus recorded an improvement in these parameters.

**Abbreviations**
a, b and c: significant differences between the groups at the threshold of 5% or 1%
ALH: amplitude of lateral head displacement (µm)
BCF: frequency at which the head crosses the midpoint of the trajectory (Hz)
CASA: computer assisted semen analysis
G c: group of rams neither stressed nor received essential oil of thym
G he: group of rams not stressed with the same doses as the (G s‐he) group
G s: group of rams heat stressed by applying heat bags to both testicles of six rams
G s‐he: group of rams heat stressed but received orally 100 µL/day/animal of thyme essential oil
HCT: haematocrit
Hgb: haemoglobin
IM: % of immobility
LIN: curvilinear path linearity (VSL/VCL ratio) (%)
MAD: mean angular displacement (°)
MCH: mean corpuscular haemoglobin
MCHC: mean corpuscular haemoglobin concentration
MCV: mean corpuscular volume
MPR: motility progressive
MPV: platelet average size
Mtot: total motility (%)
NP: % of nonprogressive spermatozoa
PCT: plateletcrit
PDW: platelet distribution width
PLT: plate
*p* < 0.05: significant
RBC: red blood cell
RDW‐CV: red cell distribution width‐coefficient of variation
RDW‐SD: red cell distribution width‐standard deviation
STR: average path straightness or linearity (VSL/VAP) (%)
VAP: mean path velocity (µm/s)
VCL: curvilinear velocity (µm/s)
VSL: linear velocity (µm/s)
WOB: oscillation of the actual trajectory with respect to the mean trajectory (VA/VCL) (%)

## 1 Introduction

Heat stress severely impairs sheep reproduction and poses a significant risk to the efficiency of animal production under current climatic conditions, with impacts that increase as global temperatures rise. Ruminant fertility is of economic importance and its improvement is a priority in livestock production to ensure its sustainability. High temperatures recorded in areas with hot summers affect livestock fertility (Kumar et al. 2017). In animals, heat stress (HS) triggers a series of physiological, metabolic, endocrine and molecular adaptations in the body to maintain normothermia and survive (Barragán Sierra et al. 2021; Hamilton et al. 2016). However, some of these changes adversely affect fertility, mainly endocrine. HS in rams, through various mechanisms, causes a decrease in testosterone levels in the blood and adversely affects the processes of spermatogenesis and sexual behaviour. HS is a detrimental factor in many biological systems in the body, including the circulatory system, skin system, respiratory system and male reproductive activity (Boni 2019). High temperatures lead to increased testicular metabolism and sperm damage. Oxidative stress is the main cause of testicular damage caused by HS. Testicles are organs of the male reproductive tract that are involved in spermatogenesis. In mammals, testis temperature must be 2°C–8°C below body temperature to ensure successful spermatogenesis (Nguyen‐Thanh et al. 2022; Jung, Eberl, and Schill 2001). Chronic HS is a risk factor affecting the reproductive system (Kanter and Aktas 2009). Higher temperatures lead to increased testicular metabolism without a corresponding increase in blood supply, leading to local hypoxia and detrimental tissue effects (Reyes et al. 2012). A phenomenon known as hypoxia‐reperfusion injury can occur (Ngoula et al. 2017; Hou et al. 2012). In this condition, an oxidative imbalance can occur after the restoration of normal temperature and tissue reperfusion. This situation is explained in studies in which suppression of testicular function under heat stress led to reduced fertility in ruminants (Belhadj Slimen et al. 2019), HS can adversely affect ram reclamation through several mechanisms. The main ones are reduced testosterone concentrations and direct damage to sperm morphometry and genetic material content (Binsiya et al. 2017; Damián, Bausero, and Bielli 2015). This is reflected in disturbances in the process of spermatogenesis, as well as reduced semen quality, reduced reproductive behaviour and reduced fertility (Moura et al. 2019; Kahwage et al. 2018). However, the implementation of HS mitigation strategies improves ram fertility in these climatic conditions. Therefore, using HS mitigation strategies in rams is necessary to maintain herd fertility, especially during the hot season of hot weather years. Antioxidant responses to stressful events in acute situations may involve immediate responses achieved primarily through protein activation. On the other hand, long‐term responses that require gene activation and translation of new proteins are also important (Rahal et al. 2014). To mitigate heat stress‐induced oxidative stress, it is speculated that feeding with natural antioxidants can be used as a curative and therapeutic strategy to prevent oxidative stress induced by heat stress (El‐Hanoun et al. 2014). Thyme (*Thymus vulgaris*) is known to improve antioxidant activity (Pandur et al. 2022; Placha et al. 2013). The main components of thyme essential oil are thymol, carvacrol, p‐cymene, g‐terpinene, linalool, b‐myrcene and terpinene‐ol (Lee et al. 2005). These ingredients are known to have antioxidant properties (Rota et al. 2008). Currently, there is limited research on the effects of thyme (*T. vulgaris*) essential oil on the exocrine function of the gonadal glands in heat‐stressed rams.

In this context, this study aimed to assess the effect of tests of heat stress on the fertility of rams and to study the protective potential of a natural antioxidant source (*T. vulgaris* essential oil).

## 2 Materials and Methods

### 2.1 Plant Material and Essential Oil Extraction

*T. vulgaris* plants were freshly collected from Aïn Draham, Jendouba Governorate (North of Tunisia, the temperature average is 18.6°C and the precipitation average is 825.4 mm) in the spring season (March and April 2019). leaves were dried at room temperature and subjected to hydrodistillation for 3 h with distilled water using a Clevenger‐type apparatus. Distilled EO was dried over anhydrous sodium sulphate, filtered and stored in opaque bottles at 4°C.

### 2.2 Analysis of Phytocompounds and Antioxidant Activity

The procedures described by Palmieri et al. (2020) were adopted to analyse total phenols (TP) and total tannins (TT). Total flavonoids (TFC) were analysed as described by (Palmieri et al. 2020). Duplicate samples of the Tymus EO were tested.

The antioxidant activity of *T. vulgaris* essential oil was evaluated by different methods: ABTS radical cation scavenging activity of Thymus EO was measured using the method of Lu et al. (2007). Radical scavenging activity (DPPH) was assayed according to the method by Vahedi et al. (2018).

### 2.3 Study Area

The study was conducted at the Bou Rbiaa experimental station belonging to the Laboratory of Animal and Fodder Production of the National Institute of Agronomic Research of Tunisia (INRAT), located 25 km southwest of Tunis at latitude of 36°38′ north and a longitude of 10°17′. The experiment was conducted in August. During the whole experimental period, the animals were kept in a covered sheepfold, individually housed in boxes with an average surface area of 80 m^2^, equipped with a feed trough and exposed to natural light through several windows located on each side.

### 2.4 Animals and Treatments

This study was done on 24 rams of the Barbarine breed. They were selected based on their suitability for collection from the artificial vagina and were subjected to 10 days of adaptation to the experimental conditions and diets. Animals were exposed to uniform feeding conditions (Table 1) and experiments were approved by the Animal Ethics Committee of the National Agronomic Research Institute of Tunisia (Protocol No. 05/15). At the beginning of the experiment, the average weight was 62.5 ± 0.3 kg, while the average scrotal diameter was 31.75 ± 1.5 cm. The average age was 24 ± 6.6 months. Rams were randomly assigned into four homogeneous groups (six rams for each one) of the same average weight and scrotal circumferences were formed. Rations were distributed in two meals; the first at 9 AM and the second at 4 PM. One hundred eight adult ewes of the same breed divided into four groups of 32 ewes were used to study the fertility of rams in vivo. For all animals, the morning meal consisted of 0.6 kg of oat hay and 0.3 kg of the concentrate mixture (250 g triticale, 45 g soybean meal and 5 g CMV). The same quantities of the same ingredients were also fed in the afternoon meal. The rations are iso‐energetic based on metabolizable energy. They were formulated for a daily intake of approximately 1.5 of the maintenance metabolizable energy requirement. The diets were fed for 14 days in addition to the adaptation period (10 days). Water was always available and daily replenished. The animals were randomly separated into four groups. The experiment consisted of inducing localized heat stress in the first group of rams by applying heat bags to both testicles of six rams (G s). The second group underwent the same heat stress on the testes but received 100 µL/day/animal of a natural source of antioxidant, thyme essential oil (G s‐he) by drugs. In this experiment, we used two control groups. The positive control was not stressed but received thyme essential oil (G he) with the same doses as the (G s‐he) group. The negative control was not stressed and did not receive thyme oil (G c).

**Table 1:** Chemical composition (g/kg of dry matter) of the diet offered to animals.

Food/parameters	Oat hay	Triticale	Soybean meal
DM (%)	88	89	91.5
OM (%)	94	96.8	98.6
CP (%)	4.8	9.2	50.8
NDF (%)	79.9	30.7	26
ADF (%)	53.9	11.7	14
ADL (%)	10.25	2	2.1

Abbreviations: ADF, acid detergent fibre; ADL, acid detergent lignin; CP, crude protein; DM, dry matter; NDF, neutral detergent fibre; OM, organic matter.

### 2.5 Environmental Parameters

**Figure 1:**
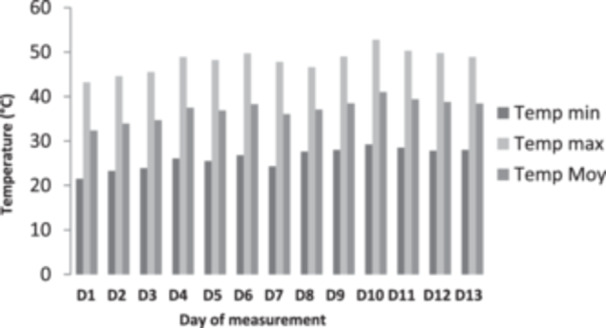
Daily temperature variation during the experiment period.

Daily recording of minimum and maximum temperatures was done with a minimum and maximum thermometer equipped with an electronic display in the building where the animals are kept. The highest temperature was 52.8°C. The lowest temperature was 21.5°C. The average temperature was 32.5°C (Figure 1).

### 2.6 Rectal and Testicular Temperatures

Rectal temperature was monitored daily throughout the experimental period using a digital thermometer applied to the rectal mucosa of the animal for 1 min. The same person carried the measurement out each time. The testicular temperature was taken daily during the whole experimental period, using infra‐red thermography (Thermal Imaging, Népenthès, France). The measurement technique consists of fixing the device at 5 cm, towards the centre of each testicle (left and right) and recording the value that appears.

### 2.7 Scrotal Circumference

The anteroposterior circumference of the scrotum was measured for all rams once a week during the whole experimental period using a flexible measuring tape placed at the maximum diameter of the scrotal sac, as described by Martínez‐Velázquez et al. (2003).

### 2.8 Semen Collection, Sperm Volume and Concentration

The males were trained to collect semen using the artificial vagina during the 2 months preceding the experiment. The collections were done in the presence of ewes induced in heat (treatment with progestagen vaginal sponge for 12 days followed by aby an injection of estradiol benzoate). During the experimental phase, ejaculation was collected once per ram per week. The collections were made on the same day and at the same time. Immediately after collection, the volume of ejaculated semen is determined in a tube graduated to 1/10th of mL. After each ejaculate, a volume of 10 µL of semen is mixed with 3990 µL of saline solution (0.9% NaCl). The sperm concentration (expressed in billions of spermatozoa per ml) is then determined using a calibrated photometer for ovine (Accucell, IMV, Paris, France) at a wavelength of 550 nm. The apparatus is calibrated, at each count beforehand, according to regression equation:

$$C=2525\;{\text{Abs}}^{2}+2565\;\text{Abs}+780\times 10^{9}/\text{mL},$$

where *C* is the concentration in millions and Abs is the absorbance.

### 2.9 Sperm Motility and Kinematic Parameters of Spermatozoa

Motility and kinematic parameters of spermatozoa were evaluated using CASA (computer assisted semen analysis). It is composed of a phase contrast microscope equipped with a camera connected to a computer station equipped with analysis software (SPERMOTEC; Alwex 2010, Germany). This set allows us to observe and evaluate the frequencies and types of movement of the spermatozoa. A drop of sperm is placed in the well of a special blade (Makler blade). The observation is done at 40× magnification, and the evaluation is done by analysing at least 10 photos taken on the whole slide.

### 2.10 Blood Sampling Collection and Analysis

Blood samples are taken every week to determine the hemogram of each animal. The blood is collected from the jugular vein in EDTA tubes of the vacutainer type 5 mL. The tube is gently shaken and placed on ice. The analysis of the samples is performed immediately after collection using an automatic haematological analyser (MINDRAY BC 2800, France).

### 2.11 Mating Management

To study the effect of different treatments on the fertility of rams in vivo, we used 128 adult ewes of the same breed divided into four groups balanced according to age and body weight. Ewes received the same diet that covers the nutritional needs in the reproduction physiological stage. Ewes were synchronized to oestrus using intravaginal progesterone pessaries impregnated with 40 mg fluorogestone acetate and left in place for 13 days. Mating was done by bringing in rams after removing the pessary, each group of rams is subject to the breeding of 32 females. Ewes observed in oestrus were manually mated twice: Immediately after detection of oestrus and 12 h later. After lambing, the fertility and reproductive activity obtained in the first oestrus were recorded.

### 2.12 Statistical Analysis

Data obtained from this study were analysed using the SAS software (SAS version 9.4, SAS Inst., Cary, NC, USA). Data were tested for normality and variance under the assumption of homogeneity. All values were grouped, and the mean and standard error were calculated. A one‐way analysis of variance by a fully randomized design using the general linear model was performed for all parameters to examine differences between groups. Differences between LS means were assessed by Tukey–Kramer's test and *p* < 0.05 was considered a significant value. The statistical model included treatment effect, block and random error as follows:

Yijk=μ+block+Ei+εijk,

where Yijk is the observation ‘*k*’ at four treatment *E* (G c; control, G he, G s‐he and G s); *μ* is the overall mean; block is the effect of blocking, Ei are treatments and εijk is the effect of random error.

## 3 Results

### 3.1 Bioactive Compounds Content and Antioxidant Activity of *T. vulgaris* EO

Total phenolic (TPC), total flavonoid (TFC) content and antioxidant activity (DPPH, ABTS) data are reported in Table 2. The *T. vulgaris* essential oil phytochemical assays showed high concentrations of bioactive compounds in the form of antioxidants with, respectively, total phenolic and flavonoid contents of 187.3 ± 10.9 and 78.83 ± 12.17 mg of garlic acid equivalent (GAE). the antioxidant activity of the essential oil of thyme (*T. vulgaris*) is well shown by the two methods used (DPPH: 159.8 ± 16.7 and ABTS: 132.4 ± 13.9 µmol Trolox equivalent [TE]/g).

**Table 2:** Bioactive compounds and antioxidant capacity of *Thymus vulgaris* essential oil.

Component	Composition (mean ± SE)
TPC (mg GAE/g)	187.3 ± 10.9
TFC (mg GAE/g)	78.83 ± 12.17
DPPH (µmol TE/g)	159.8 ± 16.7
ABTS (µmol TE/g)	132.4 ± 13.9

*Note:* Data are represented as mean ± SE of three measurements.

Abbreviations: ABTS, 2,2′‐casino‐bis(3‐ethylbenzothiazoline‐6‐sulphonic acid); DPPH, 2,2‐diphenyl‐1‐picrylhydrazyl; GAE, gallic acid equivalent; TE, Trolox equivalent; TFC, total flavonoid content; TPC, total phenolic content.

### 3.2 Rectal Temperature

**Figure 2:**
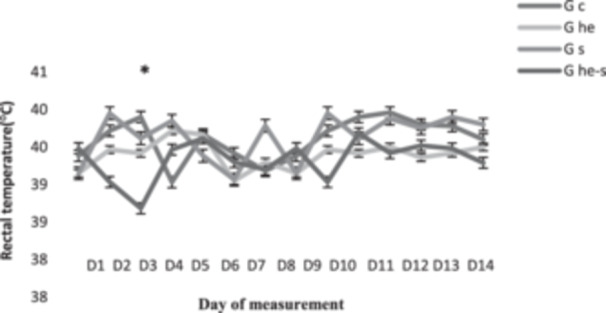
The effect of induced tests heat stress and treatment with *Thymus vulgaris* EO on fluctuations in the rectal temperature (°C) of animals. * indicate significant differences between groups at the 5%.

Results of the descriptive statistics of the rams' rectal temperature (RT) registered during the trial period are shown in Figure 2. RT monitoring showed fluctuations in all rams between 38.5°C and 40°C. The average rectal temperature of the rams on the first day of the experiment was 39.29 ± 0.14°C. We noticed that the rams that received essential oil during the stress period had lower temperatures than the stressed group (*p* < 0.05). Thus, the EO ensures the homoeostasis stability of the animal in case of stress and decreases the rectal temperature.

### 3.3 Haematological Profile

This study investigated the effect of tests heat stress and treatment with *T. vulgaris* EO, on the haematology parameters in sheep. The laboratory analysis of the blood of each group of rams is summarized in Table 3. The changes in RBC count were not significant (*p* > 0.05), throughout the periods of the experiment. Results show that the haemoglobin levels, red blood cell (RBC) number, haematocrit, mean corpuscular volume (MCV), mean corpuscular haemoglobin (MCH) content, monocytes, eosinophil and platelet counts were not affected (*p* > 0.05) by tests heat stress and the treatment with *T. vulgaris* EO. However, mean corpuscular haemoglobin concentration (MCHC), was affected by *T. vulgaris* EO supplementation (*p* < 0.05).

**Table 3:** The effect of tests heat stress and treatment with *Thymus vulgaris* EO on the haematological profile.

Parameter	Treatment				SEM
	G c	G he	G s	G s‐he	
RBC (10^12^/L)					
Day 0	8.43	7.95	6.38	7.71	
Day 7	8.26	7.92	8.31	8.27	1.29
Day 14	8.24	7.98	7.69	8	
Average	8.31a	7.95a	7.46a	8.00a	
HGB (g/dL)					
Day 0	10.08	10.53	11.3	10.38	
Day 7	10.83	10.58	11.73	11.3	2.08
Day 14	12.33	10.91	10.73	11.78	
Average	11.08a	10.67a	11.25a	11.15a	
HCT (%)					
Day 0	32.51	35.5	31.23	33.2	
Day 7	34.18	33.38	36.58	33.91	6.7
Day 14	34.05	34.81	33.7	35.33	
Average	33.58a	34.56a	33.84a	34.15a	
MCV (fL)					
Day 0	40.86	42.51	42.76	41.6	
Day 7	41.36	42.2	43.31	41.15	11.08
Day 14	41.33	43.61	43.7	41.76	
Average	41.18a	42.77a	43.26a	41.50a	
MCH (pg)					
Day 0	12.63	12.63	13.15	13.03	
Day 7	13.06	13.35	13.88	13.65	3.08
Day 14	13.33	13.68	14.01	13.9	
Average	13.01a	13.22a	13.68a	13.53a	
MCHC (g/L)					
Day 0	309.83	297.83	306.5	263.81	62.9
Day 7	316.66	317.5	268.6	332.83	
Day 14	323.16	314.33	271.06	333.83	
Average	316.55a	309.89a	282.05b	310.16a	
RDW‐CV (%)					
Day 0	16.55	16.11	17.36	16.18	
Day 7	16.76	15.86	17.66	16.1	5.1
Day 14	17.16	16.21	17.61	16.46	
Average	16.82a	16.06a	17.54a	16.25a	
RDW‐SD (fL)					
Day 0	24.18	24.18	26.25	23.78	
Day 7	24.43	23.6	26.98	23.43	3.6
Day 14	24.93	24.91	27.08	24.26	
Average	24.51a	24.23a	26.77a	23.82a	
PLT (10^9^/L)					
Day 0	364.5	275	303.5	365.66	
Day 7	301.5	327.33	349.66	352	88.4
Day 14	264.83	293.66	353	231.5	
Average	310.28a	298.66a	335.39a	316.39a	
MPV (fL)					
Day 0	6.31	6.28	6.35	6.18	
Day 7	6.15	6.05	6.11	6	0.85
Day 14	6.18	6.1	6.2	6.15	
Average	6.21a	6.14a	6.22a	6.11a	
PDW					
Day 0	15.01	15.25	15.03	14.91	
Day 7	15.1	15.08	14.86	15	2.9
Day 14	15.05	15.13	15	15.16	
Average	15.05a	15.15a	14.96a	15.02a	
PCT (%)					
Day 0	0.22	0.1195	0.19	0.27	
Day 7	0.18	0.19	0.21	0.21	0.001
Day 14	0.16	0.17	0.21	0.14	
Average	0.19a	0.16a	0.20a	0.21a	

*Note:* Letters a, b, c, and d in the table indicate significant differences between groups at the 5% threshold.

Abbreviations: HCT, haematocrit; HGB, haemoglobin; (VSL/VCL ratio) (%); MCH, mean corpuscular haemoglobin; MCHC, mean corpuscular haemoglobin concentration; MCV, mean corpuscular volume; MPV, platelet average size; PCT, plateletcrit; PDW, platelet distribution width; PLT, plate; RBC, red blood cell; RDW‐CV, red cell distribution width‐coefficient of variation; RDW‐SD, red cell distribution width‐standard deviation.

### 3.4 Testicular Temperature

**Figure 3:**
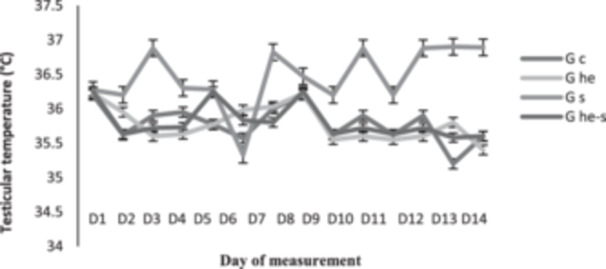
The effect of tests heat stress and treatment with *Thymus vulgaris* EO on variations in the daily temperature (°C) of testicles.

Figure 3 shows the evolution of the daily testicular temperature in rams of the different groups during the period of the trial. The average testicular temperature of the rams on the first day of the experiment was 36.23 ± 0.03. Exposure of the animals to heat stress in the testicles caused an increase in testicular temperature with peaks on Days 3, 7 and 11 followed by a plateau until Day 14 (*p* > 0.05). In addition, it was observed that the stressed animals had a higher temperature on Day 4 compared to the other groups (*p* < 0.05).

### 3.5 Scrotal Circumference

**Figure 4:**
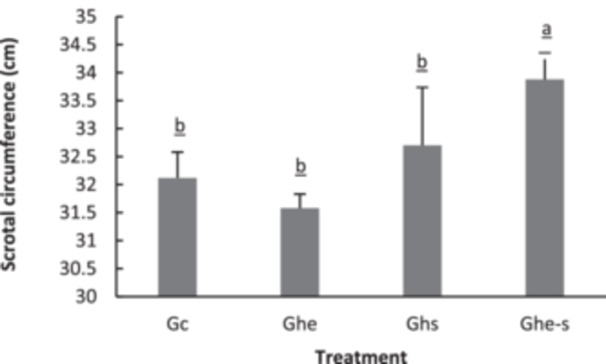
The impact of induced heat stress and treatment with *Thymus vulgaris* EO on changes rams' scrotal circumference (measured in centimeters). Letters a, b and c in the table indicate significant differences between groups at the 5% threshold. G c, control group; G he, a group of rams not stressed with the same doses as the (G s‐he) group; G s, a group of ram's heat stressed by applying heat bags to both testicles of six rams; G s‐he, a group of ram's heat stressed but received orally 100 µL/day/animal of thyme essential oil.

The effects of thymus EO on the evolution of Scrotal circumference are shown in Figure 4. Results showed that scrotal circumference increased significantly (*p* < 0.01) in the G s‐he group as compared to other groups. At the beginning of the experiment, the average scrotal circumference in the four groups of rams was 32 ± 1.2 cm. However, there was a significant increase in this parameter up to 33.7 cm, compared to the other groups. This probably shows the protection of the testes against the stressful environment by increasing the scrotal circumference.

### 3.6 Sperm Parameters

The average values of the sperm parameters of each group of rams are presented in Table 4. Analysis of sperm parameters showed no effect of heat stress on volume (*p *> 0.05). However, the results showed a decrease in sperm concentration G s‐rams with a value of 5420.61 × 10^9^/mL (*p* > 0.05). As represented in Table 3, rams in the (G s‐he) group were similar to the control group (G c) (*p* > 0.05). Total sperm motility decreased with heat stress to 76.18%; however, it increased in the G he and G s‐he groups, respectively, 84.42, and 82.37% (*p* < 0.05). On the other hand, the immobility of the spermatozoa of the stressed animals increased when compared with the control group (G c = 18.17%) in contrast to the group treated with EO (15.56% and 15.56%, respectively, for G he and G s‐he. However, the sperm normal morphology was improved significantly in all treated *T. vulgaris* essential oils groups (*p* < 0.05).

**Table 4:** Effect of heat stress and *Thymus vulgaris* EO treatment on Semen parameters.

Parameters	Treatments				
	G c	G he	G s	G s‐he	SEM
Volume (mL)					0.14
Day 0	1	1.06	0.95	1	
Day 7	1.1	1.05	0.91	1.06	
Day 14	0.95	1.01	0.96	1.03	
Average	1.01a	1.04a	0.94a	1.03a	
Concentration (10^9^/mL)					532.1
Day 0	5654	5245.66	5273.83	5566.66	
Day 7	5664.83	5512.83	5555	5999.83	
Day 14	6323.66	6271.16	5433	5578.5	
Average	5880.83a	5676.55a	5420.61a	5714.99a	
MPR (%)					5.2
Day 0	29.55	30.04	30.085	29.01	
Day 7	30.50	31–30	26.61	31.91	
Day 14	33.62	36.27	31.68	32.04	
Average	31.22a	32.53a	29.45a	30.98a	
NP (%)					9.2
Day 0	43.63	43.5	42.69	43.04	
Day 7	51.23	54.14	49.21	54.50	
Day 14	56.92	58.01	48.27	56.61	
Average	50.59a	51.88a	46.72a	51.38a	
Mtot (%)					12.8
Day 0	73.19	73.54	72.77	72.05	
Day 7	81.73	85.45	75.82	86.41	
Day 14	90.54	94.29	79.96	88.66	
Average	81.82a	84.42a	76.18b	82.37a	
IM (%)					8.8
Day 0	26.81	26.45	27.22	27.94	
Day 7	18.26	14.55	24.17	13.58	
Day 14	9.45	5.70	20.03	11.33	
Average	18.17b	15.56c	23.80a	17.61b	
VSL (µm/s)					
Day 0	5.05	4.54	4.51	4.74	0.8
Day 7	5.38	5.35	6.36	6.17	
Day 14	5.60	6.45	5.73	5.07	
Average	5.34a	5.44a	5.53a	5.32a	
VCL (µm/s)					
Day 0	14.15	13.74	12.27	16.16	6.1
Day 7	16.16	15.9	20.55	19.82	
Day 14	16.16	19.75	17.43	14.17	
Average	15.49a	16.46a	16.91a	16.71a	
VAP (µm/s)					
Day 0	9.61	9.01	8.80	8.48	
Day 7	10.05	9.97	12.49	11.89	0.32
Day 14	10.12	12.35	10.73	9.02	
Average	9.92a	10.44a	10.67a	9.79a	
LIN (%)					
Day 0	37.28	39.02	39.88	38.71	
Day 7	35.40	36.59	32.74	32.63	7.6
Day 14	37.25	37.70	35.35	38.57	
Average	36.64a	37.77a	35.99a	36.63a	
WOB (%)					
Day 0	65.02	66.44	65.02	67.04	
Day 7	63.77	65.93	61.39	60.92	10.02
Day 14	64.08	68.04	62.87	65.3	
Average	64.29a	66.80a	63.09a	64.42a	
STR (%)					
Day 0	56.38	58.53	58.84	56.26	
Day 7	55.24	55.26	52.92	53.34	14.2
Day 14	57.69	53.27	55.89	58.59	
Average	56.43a	55.68a	55.88a	56.06a	
MAD (°)					
Day 0	27.05	26.21	24.91	26.24	
Day 7	35.60	28.05	31.85	37.04	6.08
Day 14	20.11	32.60	29.54	25.94	
Average	27.58a	28.95a	28.76a	29.74a	
ALH (µm)					
Day 0	3.91	3.53	3.59	3.63	1.22
Day 7	4.65	4.35	4.03	5.25	
Day 14	2.55	3.82	3.67	3.24	
Average	3.70a	3.9a	3.76a	4.04a	
BCF (Hz)					
Day 0	1.45	1.49	1.44	1.41	0.20
Day 7	1.63	1.52	1.82	2.09	
Day 14	1.16	1.57	1.50	1.30	
Average	1.41a	1.52a	1.58a	1.6a	

*Note:* Letters a, b, c and d in the table indicate significant differences between groups at the 5% threshold.

Abbreviations: ALH, amplitude of lateral head displacement (µm); BCF, frequency at which the head crosses the midpoint of the trajectory (Hz); IM, % of immobility; LIN, curvilinear path linearity (VSL/VCL ratio) (%); MAD, mean angular displacement (°); MPR, motility progressive; Mtot, total motility (%); STR, average path straightness or linearity (VSL/VAP) (%); VAP, mean path velocity (µm/s); VCL, curvilinear velocity (µm/s); VSL, linear velocity (µm/s); WOB, oscillation of the actual trajectory concerning the mean trajectory (VA/VCL) (%).

### 3.7 Reproductive Performance of Ewes After Natural Fertilization With the Different Groups of Rams

Results for the effects of supplementation treatments on the reproductive performance of each group of ewes are summarized in (Table 5). The treatments have a significant effect on all reproductive parameters of ewes. The groups were supplemented with herbal essential oil. and the control group had higher fertility (*p* < 0.05) as compared to the stressed groups. Also, we have reported a higher Heat Return rate in the stressed group (40.63%).

**Table 5:** The effect of tests heat stress and treatment with *Thymus vulgaris* EO on in vivo ram's fertility.

	G c	G he	G s‐he	G s
Oestrus (%)	100a	100a	100a	100a
Heat return (%)	21.88a	18.75a	15.63a	40.63b
Fertility (%)	66.67a	68a	60.87a	56b
Prolificity (%)	100a	100a	100a	100a

*Note:* Letters a, b, c and d in the table indicate significant differences between groups at the 5% threshold.

## 4 Discussion

Tunisian Barbarin sheep are known for their spectacular resistance to extreme stress situations (Khnissi et al. 2014, 2015) by several physiological mechanisms probably due to the significant reserves of fat that they possess and the specificity of their fatty tail. In this study, we confirmed this hypothesis, and rams were able to withstand stress caused by exposure of the testicles to heat during the summer season which naturally coincides with the reproduction season and they were able to preserve their fertility, despite the recorded drop in some parameters of sperm quality which negatively affected the fertility of rams in vivo. Monitoring of the state of health of the animals throughout the experiment is carried out by several daily measurements: rectal temperature and testicular temperature. and weekly measurements: monitoring of complete blood count (CBC).

The range of variation of rectal temperature obtained in this experiment is comparable to that cited in the bibliography. According to several authors (Fahmy 1994; Shafie et al. 1994). Rectal temperature in the ovine species can vary from 38.3°C to 39.9°C under conditions of thermal neutrality. it is from 42°C that this temperature is considered dangerous. The averages observed in the Barbarine breed from the trial are not far from the values measured in other breeds adapted to hot and arid climates. In hot weather, rectal temperatures averaged 39.7°C for Omani sheep (Srikandakumar, Johnson, and Mahgoub 2003) and 39.5°C for the Awassi fat‐tailed breed highly adapted to semi‐arid climate (Turner and Lysiak 2008) and 39.3°C for the Egyptian Rahmani breed (Hamadeh et al. 1997). In contrast. under the same climatic conditions. the rectal temperature averaged 40.6°C for the Suffolk breed (Hamadeh et al. 1997) and 40.2°C for the French island (El‐Sheikh, Ibrahim, and Salem 1981).

High testicular temperature affects spermatogenesis it causes sterility by causing abnormalities in the germ cells. Therefore, stress increases the testicular temperature which can cause infertility. Heat stress increases the testicular temperature which can affect ram fertility. Protection of the testes from the external environment is provided by the scrotum. This increases its circumference in stressful situations such as heat exposure (Marai et al. 2007). Indeed, the increase in scrotal circumference recorded in rams exposed to heat stress can be considered an adaptive or counteractive mechanism to the stressor. Heat stress caused the decrease in testicular weight. Similar to our result (Rocha et al. 2015) showed that mice subjected to hyperthermia in the tests revealed a significant reduction in testicular weight.

The observed values for all haematological values were within the range given to ram lambs by (Sanaz et al. 2021; Lepherd et al. 2009). However, studies by (Akanmu, Hassen, and Adejoro 2020) and (Jiwuba et al. 2017) showed the supplementation of Moringa extracts, to goat diets increased white blood cell counts but did not affect lymphocyte counts. Contrary to the observed responses in this study, *T. vulgaris* EO tested as an anti‐methampenogen additive did not adversely affect the blood profile of rams, a parameter indicative of animal health status. This means that it is justified for use as an additive in foods. The changes in haematological parameters in rams after induction of heat stress in the testes and/or by oral treatment with Thyme essential oil were nonsignificant (*p* > 0.05) compared to the control group. The essential oils of *T. vulgaris* used in this experiment have higher antioxidant activity compared to the findings of previous studies (Olugbenga, Gbadebo, and Adeoye 2014; Gedikoğlu, Sökmen, and Çivit 2019). Several studies have reported that thyme is a source rich in bioactive compounds (Delgado et al. 2014; Golkar, Mosavat, and Jalali 2020). A comparison with the literature shows that our data are consistent with those of various authors (Fayad et al. 2013; Moccia et al. 2020). According to the same authors, *T. vulgaris* EO has higher antioxidant activity.

Scrotal circumference increased significantly in stressed rams treated with thyme EO. This result shows that under treatment with thyme EO the testicles of these animals developed a protective mechanism by increasing scrotal circumference. A study by (Sena, Dantas, and Pereira 2018) on the effect of *Cochlospermum planchonii* rhizome powder on testicular morphometry and semen quality in West African Dwarf goats found that dietary inclusion of *C. planchonii* rhizome powder had a significant effect on testicular morphometry and semen quality. thus. an increase in total sperm count, concentration and motility was noted. This conclusion thus corresponds with Mhomga et al. (2019) and Ray et al. (2014), who reported that the use of plant extracts improves scrotal homoeostasis and prevents testicular cell death, thereby enhancing male fertility. In addition, an improvement in fertility, and an increase in testicular size, scrotal circumference, sperm concentration and viability have been noted by Syarifuddin et al. (2017) and Tahvilzadeh et al. (2016) further explained that antioxidant components such as flavonoids, terpenoids, saponins, tannins, steroids and cardiac glycosides from *C. planchonii* positively influence the reproductive index of livestock.

These results are consistent with those reported by (Abu, Ochalefu, and Ibrahim 2012), who reported that plant extracts can enhance peripheral testosterone and sperm motility. Other work has shown no effect of high temperatures on spermatogenesis in native Indian sheep breeds. On the other hand, exposure of local Creole billy goats from Guadeloupe to direct sunlight does not affect their semen quality (Ebenebe, Okoli, and Ogbi 2017). The treatment of heat‐stressed animals with Thyme essential oil ensured an increase in testicular weight. Several studies have reported the beneficial effects of extracts from medicinal plant species as herbal antioxidants. According to the study of (Baril, Chemineau, and Cognie 1993), the incorporation of fresh leaves of wormwood and/or rosemary in the diet of rams had varying effects on semen characteristics. However, some authors report no effect of antioxidant supplementation on semen volume and sperm concentration in rams (Khnissi et al. 2023; Cofré‐Narbona et al. 2016). In the current study, we showed that the addition of *T. vulgaris* extract improved gross and progressive motility, and velocity parameters *T. vulgaris* essential oil probably affects these reproductive parameters as described above due to the presence of chemical compounds, which are a good source of nutrients. proved that stress negatively affects spermatozoa by causing an oxidative imbalance. Similar results have been reported by many authors (Garcia‐Oliveros et al. 2020; Gaznee, Kohli, and Kumar 2023). Studies have shown that extracts from plants have a positive effect on animals' semen (Ozer Kaya et al. 2020; Swelum et al. 2021) the in vivo study of the fertility of rams showed a decrease following heat stress and an improvement after treatment with thymus EO. Indeed, there was a drop in females fertilized on the first oestrus in rams that suffered an increase in testicle temperature and there was a high rate of return to oestrus. on the other hand, the stressed rams that received the treatment of EO of the thymus recorded a better fertility rate comparable to the control group, this result shows that treatment with EO thymus was able to reduce the harmful effects of heat stress. The in vivo fertility study was in line with the results obtained by in vitro sperm evaluation.

## 5 Conclusion

Haematology of the rams showed resistance of animals of the Barbarine breed to this stressful situation. These animals maintained their fertility during the period of stress by preserving spermatogenesis. The treatment of stressed animals with a source of natural antioxidant (*T. vulgaris*) showed the beneficial effect of the treatment on the quality of the sperm and the health of the animal during the period of stress. In Tunisia, strategies to combat heat stress are poorly developed. However, in the current context of global warming, it is important to implement devices to combat thermal stress (fans, Misters, etc.) to limit its effect on health, well‐being and performance of animals, mainly during critical physiological stages of production, like the breeding season. It was concluded that the administration of thyme extracts resulted in improved sexual performance and sperm quality of rams. Many studies have provided enough evidence to declare medicinal plants as a solution to livestock infertility. Medicinal plants are capable of alleviating sexual dysfunction and may provide a solution to poor reproductive performance in livestock due to their antioxidant and antimicrobial activities. It would be interesting to continue this study by evaluating the effects of heat stress with more in‐depth studies such as more advanced sperm analysis techniques that show proteomic and epigenetic changes in testicular tissues and evaluating the protective effects of the *T. vulgaris* with other doses.

## Conflicts of Interest

The authors declare no conflicts of interest.

## Data Availability

The data that support the findings of this study are available from the corresponding author upon reasonable request.
